# Magnifying Endoscopy with Narrow Band Imaging to Determine the Extent of Resection in Transoral Robotic Surgery of Oropharyngeal Cancer

**DOI:** 10.1155/2014/604737

**Published:** 2014-12-10

**Authors:** Ichiro Tateya, Seiji Ishikawa, Shuko Morita, Hiroyuki Ito, Tatsunori Sakamoto, Toshinori Murayama, Yo Kishimoto, Tomomasa Hayashi, Makiko Funakoshi, Shigeru Hirano, Morimasa Kitamura, Mami Morita, Manabu Muto, Juichi Ito

**Affiliations:** ^1^Department of Otolaryngology-Head and Neck Surgery, Graduate School of Medicine, Kyoto University, Sakyo-ku, Kyoto 606-8507, Japan; ^2^Department of Gastroenterology & Hepatology, Graduate School of Medicine, Kyoto University, Kyoto 606-8507, Japan; ^3^Otolaryngology of Medicine, Tokyo Medical University, Tokyo 160-0023, Japan; ^4^Institute for Advancement of Clinical and Translational Science, Kyoto University Hospital, Kyoto 6068507, Japan; ^5^Department of Clinical Oncology, Graduate School of Medicine, Kyoto University, Kyoto 6068507, Japan

## Abstract

Transoral robotic surgery (TORS) is a less invasive treatment that is becoming popular all over the world. One of the most important factors for achieving success in TORS is the ability to determine the extent of resection during the procedure as the extent of resection in the laryngopharynx not only affects oncological outcomes but also directly affects swallowing and voice functions. Magnifying endoscopy with narrow band imaging (ME-NBI) is an innovative optical technology that provides high-resolution images and is useful in detecting early superficial pharyngeal cancers, which are difficult to detect by standard endoscopy. A 55-year-old male with superficial oropharyngeal cancer has been successfully treated by combining MB-NBI with TORS and MB-NBI was useful in determining the extent of resection. ME-NBI with TORS will make it possible to achieve a higher ratio of minimally invasive treatment in pharyngeal cancer.

## 1. Introduction

Transoral robotic surgery (TORS) [1–3] is a less invasive treatment that is becoming popular all over the world. TORS has great advantages over the conventional open surgery, especially in functional outcomes such as swallowing and use of voice, and is creating a paradigm shift in the treatment strategy of laryngopharyngeal cancer.

One of the most important factors for achieving success in TORS is the ability to determine the extent of resection during the procedure as the extent of resection in the laryngopharynx not only affects oncological outcomes but also directly affects swallowing and voice functions.

Narrow band imaging (NBI) is an innovative optical technology that can increase contrast of the precise morphological changes in the mucosal surface. We [4–6] have previously reported that magnifying gastrointestinal endoscopy with NBI function (ME-NBI) provides high-resolution images and is useful in detecting early superficial pharyngeal cancers, which are difficult to detect by standard endoscopy.

In this report, we present the first case of oropharyngeal cancer that has been successfully treated by combining MB-NBI with TORS in order to estimate the precise extent of the tumor and to determine the extent of resection during the procedure.

## 2. Case Presentation

A superficial tumor lesion in the tongue base was found in a 55-year-old male during upper gastrointestinal endoscopy with a ME-NBI (GIF-H260Z, Olympus, Tokyo, Japan) as a follow-up examination after endoscopic treatment of esophageal cancer. A pathological diagnosis of the biopsy specimen revealed squamous cell carcinoma and he was referred to the Department of Otolaryngology.

**Figure 1:**
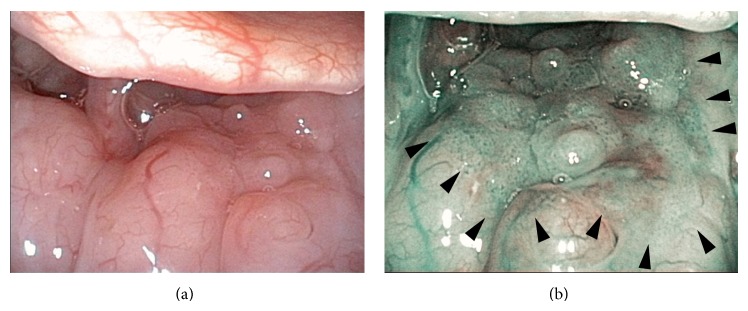
Preoperative endoscopic findings in the superficial tongue base cancer with an otolaryngological videoendoscope with NBI (ENF-type VQ). The lesion in the tongue base is hardly recognizable under white light (a). NBI visualizes the lesion as a brownish area with scattered brownish dots (arrow heads).

The superficial cancer lesion, about 1.5 cm in diameter, was identified in the left side of the tongue base with an otolaryngological videoendoscope with NBI (ENF-type VQ) (Figure 1). NBI clearly visualized the lesion as a brownish area with scattered brownish dots whereas the lesion was hardly recognizable under white light. The cancer lesion was not recognizable by computed tomography (CT), magnetic resonance imaging (MRI), or positron emission tomography (PET). Neither neck lymph node metastasis nor distant metastasis was found on CT, MRI, or PET. The lesion was clinically diagnosed as T1N0M0.

**Figure 2:**
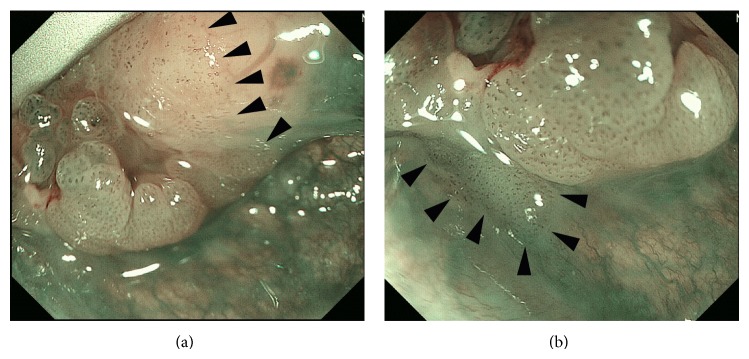
Intraoperative evaluation of the extent of the lesion with ME-NBI in the medial superior (a) and inferior (b) borders of the tumor located in the left side of the tongue base. The quality of the image is better than that of the otolaryngological videoendoscope (Figure 1) and the boundary (arrow heads) of the superficial cancer is easily traceable.

**Figure 3:**
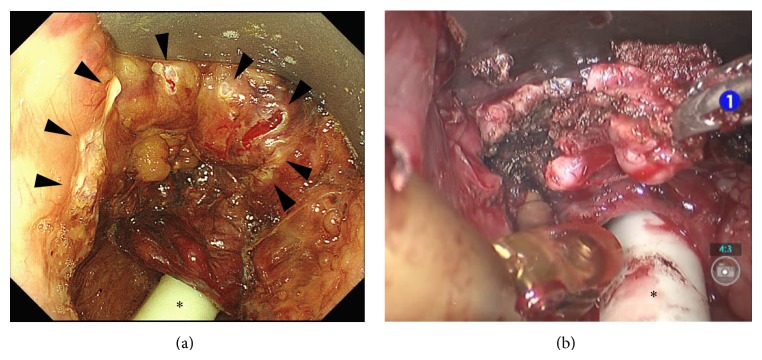
Marking dots (arrow heads) were made to indicate resection based on the findings by ME-NBI (a) and the TORS procedure with the da Vinci S surgical system (b). ∗: intubation tube.

Tongue base resection with TORS was performed under general anesthesia by cooperating with gastroenterologists. Following transnasal intubation, the FK-WO laryngoscope was inserted to visualize the surgical field. ME-NBI was then inserted transorally and the lesion was observed to confirm a boundary (Figure 2). The main tumor was slightly elevated, with a flat lesion spread surrounding the elevated area. ME-NBI visualized the flat lesion as a brownish area with scattered dots of abnormal vessels, which were invisible in white light. The boundary of the tumor was easily traceable with ME-NBI. The extent of the lesion was confirmed by iodine staining. Then, marking dots were made surrounding the boundary of the lesion with an electric needle knife (Figure 3). An incision line at the lower boundary of the lesion was made with the electric needle knife and the procedure was switched to TORS. The horizontal safety margin was about 5 mm.

The da Vinci S surgical system (Intuitive Surgical Inc., Sunnyvale, CA, USA) with three arms was used for the TORS procedure. The tumor was resected with two instrument hands, 8 mm Maryland dissector, and 8 mm monopolar cautery under the vision of the 3D endoscope (Figure 3). The resection started from the superior side and a branch of the lingual artery was ligated with clips. The depth of the tongue base resection was about 5 mm as the tumor was superficial, and the procedure was completed after confirming a negative margin in the frozen sections. No neck dissection was performed. The fasting period was 2 days following the surgery. The pathological diagnosis was squamous cell carcinoma with subepithelial invasion, and the size was 18 mm in diameter. The final diagnosis was pT1 and the pathological margin was negative.

The patient is eating a normal diet and has no evidence of local recurrence or metastasis 6 months after the surgery.

## 3. Discussion

This is the first report on combining ME-NBI with TORS in treatment of pharyngeal cancer.

NBI is an optical image enhancement technology installed in endoscopy systems that can display the mucosal surface layer in high contrast, especially hemoglobin-rich areas such as blood vessels. Combined with magnifying endoscopy, NBI can capture the microvascular pattern on the lining of the digestive tract [7]. ME-NBI has the capabilities of both standard video endoscopy and adjustable image magnification over a continuous range up to a magnification factor of 80 [6]. It provides higher resolution images with higher contrast compared to endoscopes with NBI used in otolaryngology, such as ENF-VQ and ENF-VH (Olympus Medical Systems, Tokyo, Japan), and enables detection of early superficial laryngopharyngeal cancers, which are difficult to detect by standard endoscopy or the type of nonmagnifying endoscopy with NBI used in otolaryngology. In this case, combining ME-NBI with TORS made it possible to estimate the horizontal extent of the superficial lesion precisely, which was beneficial in determining the extent of resection.

There are two advantages in using ME-NBI for TORS. One is that the combination facilitates early diagnosis and less invasive treatment of pharyngeal cancer. An increasing number of superficial pharyngeal cancers are expected to be discovered with the increasing use of ME-NBI [8]. The concept of the superficial cancer came from the digestive tract, such as esophagus and stomach. The superficial cancers of esophagus and stomach are usually treated with either endoscopic mucosal resection (EMR) or endoscopic submucosal dissection (ESD) using a gastrointestinal endoscope. These superficial lesions in the laryngopharynx can be treated with TORS and ME-NBI will be essential during the TORS procedure because we need to “see” the lesion during the surgery. Early diagnosis of pharyngeal cancer with ME-NBI and less invasive treatment for such lesions with TORS combined with ME-NBI will contribute to progress in the treatment of pharyngeal cancer.

The other advantage of the ME-NBI TORS combination is associated with invasive cancer lesions. When observed with ME-NBI, many invasive cancers have superficial lesions surrounding invasive lesions. It is generally accepted that the safety margin needed for the resection of superficial lesion is less than that for the resection of invasive cancer in the upper digestive tract. The safety margin needed for the resection of superficial lesion is about 2 mm [9] to 5 mm [10] whereas that of invasive cancer is 10 to 15 mm. The boundary of a superficial lesion can be easily identified with ME-NBI, so the combination of ME-NBI with TORS will be beneficial in avoiding excessive resection, especially for the superficial part of the invasive cancer, thus resulting in better functional outcomes, such as swallowing and voice function.

The limitation of ME-NBI is that ME-NBI is not useful for examining deeper tissue beneath the epithelium. Frozen section may not be necessary for checking the horizontal margin of the tumor, but it is still necessary for checking the vertical margin.

## 4. Conclusions

The reported case of oropharyngeal cancer was successfully treated by combining MB-NBI with TORS and MB-NBI was useful in determining the extent of resection. ME-NBI with TORS will make it possible to achieve a higher ratio of minimally invasive treatment in pharyngeal cancer. Further study is needed to validate the benefit of combining ME-NBI with TORS.
